# Constructing Three-Dimensional Architectures to Design Advanced Copper-Based Current Collector Materials for Alkali Metal Batteries: From Nanoscale to Microscale

**DOI:** 10.3390/molecules29153669

**Published:** 2024-08-02

**Authors:** Chunyang Kong, Fei Wang, Yong Liu, Zhongxiu Liu, Jing Liu, Kaijia Feng, Yifei Pei, Yize Wu, Guangxin Wang

**Affiliations:** 1 Provincial and Ministerial Co-Construction of Collaborative Innovation Center for Non-Ferrous Metal New Materials and Advanced Processing Technology, School of Materials Science and Engineering, Henan University of Science and Technology, Luoyang 471023, China; kcy00312@163.com (C.K.); liuzhongxiu2020@163.com (Z.L.); liuliu158600@163.com (J.L.); fengkaijia2021@163.com (K.F.); 15738595833@163.com (Y.P.); 221402010224@stu.haust.edu.cn (Y.W.); 2 Faculty of Engineering, Huanghe Science & Technology University, Zhengzhou 450063, China; wangf157@hhstu.edu.cn; 3 Research Center for High Purity Materials, Henan University of Science and Technology, Luoyang 471023, China

**Keywords:** alkali metal anode, 3D Cu-based current collector, dendrite free, electrochemical performance

## Abstract

Alkali metals (Li, Na, and K) are deemed as the ideal anode materials for next-generation high-energy-density batteries because of their high theoretical specific capacity and low redox potentials. However, alkali metal anodes (AMAs) still face some challenges hindering their further applications, including uncontrollable dendrite growth and unstable solid electrolyte interphase during cycling, resulting in low Coulombic efficiency and inferior cycling performance. In this regard, designing 3D current collectors as hosts for AMAs is one of the most effective ways to address the above-mentioned problems, because their sufficient space could accommodate AMAs’ volume expansion, and their high specific surface area could lower the local current density, leading to the uniform deposition of alkali metals. Herein, we review recent progress on the application of 3D Cu-based current collectors in stable and dendrite-free AMAs. The most widely used modification methods of 3D Cu-based current collectors are summarized. Furthermore, the relationships among methods of modification, structure and composition, and the electrochemical properties of AMAs using Cu-based current collectors, are systematically discussed. Finally, the challenges and prospects for future study and applications of Cu-based current collectors in high-performance alkali metal batteries are proposed.

## 1. Introduction

With the development of society, people’s demand for energy is gradually increasing. Meanwhile, the environmental problems caused by the massive use of fossil fuels have gradually attracted people’s attention [1,2]. To overcome the above problems, new energy systems are being vigorously promoted, such as wind energy, solar energy and so on [3,4,5]. Nevertheless, such new energy sources have the characteristics of intermittence and fluctuation, which make it difficult to ensure the stability of energy transmission. This requires stable and reliable energy storage devices, such as rechargeable batteries, including lead-acid batteries, Li-ion batteries, Na-ion batteries, Zn-ion batteries and so on [6,7,8,9,10]. Among the many types of energy storage batteries, Li-ion batteries (LIBs) are widely commercialized in mobile phones, laptops, electric vehicles and other electronic devices because of their long lifespan and low self-discharge rate [11,12,13,14]. However, the practical specific energy of LIBs containing traditional graphite anodes is close to the theoretical value [15,16], and even though many efforts have been made to improve the specific energy of Li-ion batteries [17,18,19,20], they are still unable to meet people’s gradually increasing requirements [21,22]. Therefore, it is critical to develop new high-energy-density batteries [23].

Alkali metals (Li, Na and K) are deemed as the ideal anode materials for next-generation high-energy-density batteries because of their high theoretical energy densities (Li, 3860 mAh g^−1^; Na, 1166 mAh g^−1^; K, 685 mAh g^−1^) and low redox potentials (Li, −3.04 V; Na, −2.71 V; K, −2.93 V versus SHE) [16,23,24,25,26]. Nevertheless, the further application of alkali metal anodes (AMAs) is hindered by some issues with alkali metals, such as uncontrolled dendrite growth, infinite volume expansion during cycling, and the high reactivity between alkali metal anodes and electrolytes, dead alkali metals, and fragile solid electrolyte interphases (SEIs), which lead to low Coulombic efficiency (CE) and unsatisfactory cycling performance, as well as even safety hazards [16,27,28,29,30,31,32]. To date, considerable efforts have been made to address these problems, including electrolyte optimization, the construction of artificial SEIs on alkali metal anodes, introducing solid-state electrolytes, the modification of separators, host design and so on [29,33,34,35,36,37,38,39,40].

In recent years, it has been demonstrated that constructing three-dimensional (3D) current collectors can alleviate volume expansion and suppress dendrite growth, because they have sufficient space to accommodate AMAs’ volume expansion, and high specific surface areas, which could lower the local current density [30,41,42,43,44]. Through functional modification, current collectors can also provide multiple functions, which include reducing nucleation overpotential and local current density [34,45]. To date, Cu has been widely studied as a current collector due to its good conductivity and processability, and 3D Cu-based current collectors (3D Cu-based CCs) have received extensive attention for use as AMAs [45,46,47,48,49]. For example, An et al. prepared the 3D porous Cu CCs from CuZn alloy foil by the vacuum distillation method, and the electrochemical performances of lithium metal anodes (LMAs) with these 3D Cu CCs were greatly enhanced [46]. Furthermore, Li and co-workers used chemically treated Cu foam as the Na host, which achieved a stable Na cycling behavior and suppressed volume expansion upon cycling [47]. Moreover, an anode substrate obtained by chemically loading a thin layer of gold particles onto 3D Cu foam was reported by Zhang’s group [48]. This design can reduce K dendrite growth by forming stable SEI. In addition, Guo et al. summarized the application of 3D Cu-based CCs in lithium metal batteries (LMBs) [49]. Zhou et al. summarized the modification strategies of Cu CCs for LMBs [50]. Hence, developing 3D Cu-based CCs is a practical and feasible way to solve the problems encountered in the application of AMAs. Although some previous reviews on LMAs have mentioned 3D Cu-based CCs [49,50], to the best of our knowledge, critical reviews exclusively focusing on 3D Cu-based CCs for alkali metal anodes have rarely been reported.

Herein, we summarize recent progress on the application of 3D Cu-based current collectors in stable and dendrite-free alkali metal batteries (AMBs). The modification strategies of 3D Cu-based current collectors and corresponding electrochemical performances in LMBs are first reviewed. The preparation or modification methods, nano- or microstructures, and electrochemical properties of 3D Cu-based CCs based on different designs are systematically summarized and discussed in this section. Furthermore, the recent progress in relation to modified 3D Cu-based CCs in sodium metal batteries (SMBs) and potassium metal batteries (PMBs) is also summarized. Finally, we put forward the prospect of using 3D Cu-based CCs for high-performance AMBs.

## 2. 3D Cu-Based CCs for LMBs

Lithium metal batteries are considered promising candidates for use in the next generation of high-energy-density batteries. However, as mentioned above, lithium metal anodes face serious problems of dendrite growth and volume change, which hinder the practical application of LMBs. To tackle these problems, the researchers proposed using 3D CCs as lithium hosts. Meanwhile, a reasonable electrode structure can also promote the rapid transport and uniform deposition of lithium ions [51]. Due to their good electrical conductivity and processability [52], Cu-based materials have been widely studied for use in the construction and modification of 3D current collectors. To date, most of the researches on three-dimensional Cu-based CCs have focused on the three-dimensional structure design (or structural modification) and surface chemical modification of the current collectors. In this chapter, we will systematically introduce the modification strategies commonly used in three-dimensional Cu-based CCs and the corresponding electrochemical properties of Cu-based CCs in LMBs.

### 2.1. Structural Modification

Structural modification is a strategy to promote the uniform deposition of lithium ions, mitigate volume changes in lithium metal anodes, and enhance the stability of LMAs by designing or adjusting the structure of the current collector [53,54]. To date, several methods have been widely used in the preparation of three-dimensional copper-based current collectors, including the template method [55], the dealloying method [56], the electrodeposition method [57], etc.

#### 2.1.1. Template Method

The template method is an important technique used to fabricate micro- and nanostructured materials [52], especially in the preparation of porous materials. Materials with different structures can be prepared by using different templates. This method has been widely used in the preparation of 3D Cu-based CCs. Besides this, according to the types of templates, template method could be roughly divided into the inorganic template method and the organic template method.

**Figure 1:**
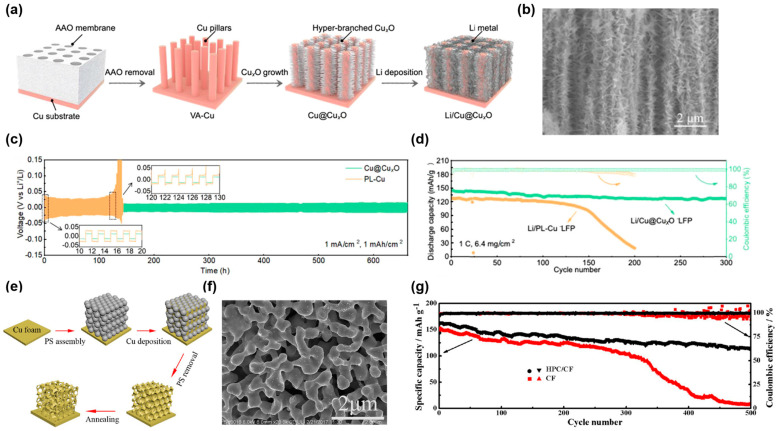
(**a**) Synthesis procedure of Cu@Cu_x_O current collector and Li/Cu@Cu_x_O electrode. (**b**) Side-view scanning electron microscopy (SEM) images of Cu@Cu_x_O. (**c**) Galvanostatic voltage profiles of Li/Cu@Cu_x_O and Li/PL–Cu symmetric cells. (**d**) Cycling performance of Li/Cu@Cu_x_O|LFP and Li/PL–Cu|LFP at 1 C. Reprinted with permission [58]. Copyright 2023, Wiley-VCH. (**e**) Schematic illustration of the synthetic procedure of the 3D HPC/CF. (**f**) SEM images of the 3D HPC/CF. (**g**) Cyclic stability of Li@3D HPC/CF|LFP and Li@CF|LFP full cell at 2 C. Reprinted with permission [60]. Copyright 2018, American Chemical Society.

The inorganic template method uses inorganic materials as templates to prepare other materials with different 3D structures. For example, He and co-workers reported 3D Cu-based CCs prepared by using NaCl as the template [43]. After the NaCl template is removed, the copper powder is successfully converted into a 3D Cu skeleton with abundant micropores. The open micrometer-sized pores and high surface area of the 3D CCs can promote the uniform distribution of Li^+^ flux and homogeneous Li plating. Consequently, the CE of Li deposition on the 3D CCs was maintained above 95% at 400 cycles at 1 mA cm^−2^. In addition, Chen et al. prepared a lithiophilic hyperbranched Cu nanostructure on Cu foil using an anodic oxide aluminum (AAO) membrane as the template [58]. Figure 1a shows the process of fabricating the CCs and the anodes. With the assistance of the AAO membrane, the vertically aligned Cu (VA-Cu) pillars were deposited on the Cu foil. After that, as shown in Figure 1b, the hyperbranched oxides were grown in-situ on the Cu pillars. The numerous lithiophilic Cu_x_O hyperbranches can act as nucleation sites, thus promoting homogeneous Li deposition. Compared with copper foil (99.2 mV), Cu@Cu_x_O exhibits a lower nucleation overpotential (44.3 mV) at 1 mA cm^−2^ and 1 mAh cm^−2^. Therefore, the Li/Cu@Cu_x_O electrodes exhibited a low and stable overpotential of 20 mV, and could maintain this over 600 cycles at 1 mA cm^−2^ and 1 mAh cm^−2^, as presented in Figure 1c. Moreover, the Li/Cu@Cu_x_O||LiFePO_4_ full cell exhibited a high specific capacity and a high-capacity retention rate of 87.6% after 300 cycles, as shown in Figure 1d. The outstanding electrochemical performances can be ascribed to the improved lithiophilicity and sufficient nucleation sites, which promote uniform Li deposition. Meanwhile, the 3D structure also contributes to cycling performance due to the effect of mitigating the electrode volume change.

In addition, some organic compounds can also be used as templates to prepare or modify 3D Cu-based CCs. For example, Stan and coworkers reported an open-porous 3D Cu-based current collector by using polylactic acid (PLA) nanoparticles as the template [59]. These 3D structures with large specific surface areas can effectively reduce local current density and nucleation overpotential. Moreover, the dendrite growth and volume change are also alleviated due to the abundance of internal space. As a result, the performance of a full battery with zero-excess lithium assembled from this current collector has been significantly improved. Similarly, Ke et al. successfully prepared highly porous copper structures on copper foam (HPC/CF) using polystyrene (PS) microspheres as the template [60]. Figure 1e shows the fabrication process of the 3D HPC/CF composite. The 3D hierarchically bicontinuous porous skeleton has numerous highly curved submicron-sized copper ligaments, which can be used as the preferred Li deposition sites, as presented in Figure 1f. The HPC/CF have a larger pore area (0.05 m^2^ g^−1^) than pristine Cu foam (0.023 m^2^ g^−1^), and the pore volume of the HPC/CF can reach 0.5216 cm^3^ g^−1^, which provides abundant internal space to accommodate the deposited lithium, relieving the volume change of the electrode. The 3D HPC/CF current collectors can effectively suppress the Li dendrites’ growth and improve the Li plating/stripping behavior. As a result, the LMAs derived from the 3D HPC/CF skeleton exhibited a high capacity for retention of 71.1% at 2 C for 500 cycles, which shows a good application prospect, as displayed in Figure 1g. The outstanding cycling performances can be ascribed to the inhibition of dendrite growth owing to the superior Li dendrite growth inhibition achieved through the novel structural design.

In summary, the 3D Cu-based CCs prepared by the inorganic template method or the organic template method have shown a good application prospect in improving the stability of LMAs. However, the reusability of templates and their suitability for large-scale preparation need to be further considered and improved.

#### 2.1.2. Dealloying Method

The dealloying method is a technique that removes some specific components from an alloy to construct 3D skeletons with interconnected channels and nanopores [61]. The dealloying method is widely used to construct 3D copper current collectors due to the adjustable porosity and ease of operation it offers [62]. At present, the main technological routes of dealloying can be divided into three categories: chemical etching [63,64], vapor dealloying [65] and electrochemical etching [66].

Chemical etching is a facile, low-cost, and controllable preparation technique. For example, Li et al. used sulfuric acid (H_2_SO_4_) and zinc sulfate (ZnSO_4_) solution to etch brass foils (Cu–Zn alloy) and prepared Cu-based CCs with 3D structures [67]. The prepared porous Cu could promote uniform Li deposition and suppress the growth of Li dendrites. The porous Cu CC showed a high and stable CE and low overpotential. Compared to planar Cu foil, the porous Cu can significantly improve the cyclic stability of LMBs.

**Figure 2:**
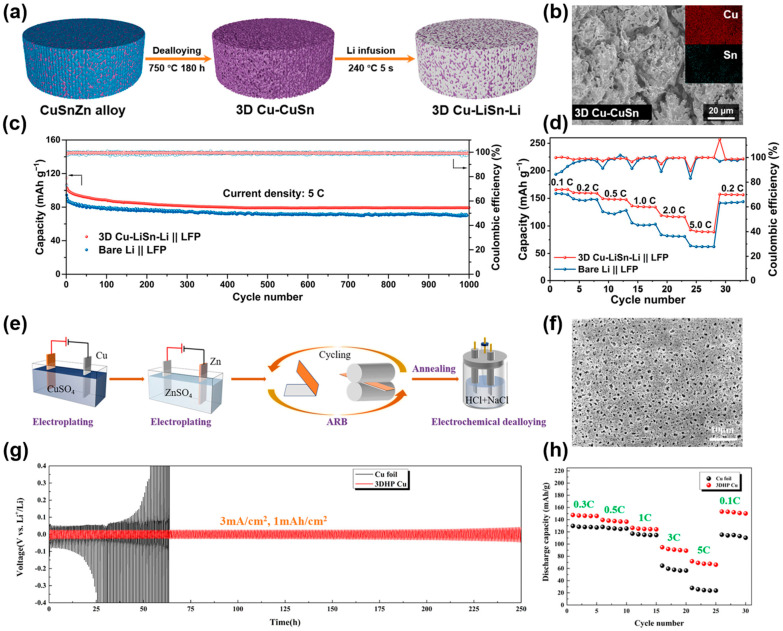
(**a**) Progress of synthesizing the 3D Cu–CuSn and 3D Cu–LiSn–Li electrodes. (**b**) Cross-sectional SEM images with the corresponding EDS elemental mapping of the 3D Cu–CuSn. (**c**) Cycling stability of the 3D Cu–LiSn–Li||LFP and Li||LFP batteries at 5 C. (**d**) Rate capabilities of 3D Cu–LiSn–Li||LFP and Li||LFP full cells. Reprinted with permission [69]. Copyright 2023, American Chemical Society. (**e**) Schematic diagram of the preparation of 3DHP Cu. (**f**) SEM image of the 3DHP Cu from the top view. (**g**) Galvanostatic cycling performance of Li@3DHP Cu electrode. (**h**) Rate capabilities of Li@Cu||LFP and Li@3DHP Cu||LFP cells. Reprinted with permission [71]. Copyright 2021, American Chemical Society.

Furthermore, based on the differences in the melting and boiling points and saturated vapor pressures of different alloy components, one or several alloy components in precursor alloy can be evaporated to construct a three-dimensional skeleton structure, which is called the vapor dealloying method [68]. For example, Qian and coworkers prepared the 3D porous Cu-based CCs via a facile vacuum distillation method from brass foils [46]. Cu CCs with different pore structures can be prepared by adjusting distillation time and temperature. The 3D porous Cu can suppress Li dendrite growth and mitigate the volume change during the Li stripping/plating process. The as-prepared Cu CCs exhibited stable CE and low overpotential, thus improving the performance of LMAs. Similarly, Wang’s group reported a lithiophilic 3D Cu–CuSn porous framework, produced via a vapor phase dealloying method [69]. Figure 2a shows the procedures of fabricating 3D Cu–CuSn and the composite anode (3D Cu–LiSn–Li). Due to the sublimation of Zn and the diffusion of Cu and Sn, many irregular holes (diameters of 2–5 μm) are produced on the surface of the 3D Cu–CuSn, as shown in Figure 2b. The corresponding elemental EDS mapping images also show that copper and tin are uniformly distributed. The 3D Cu–CuSn displays a lower nucleation overpotential of 66.7 mV than the Cu foil (96.3 mV) at 1 mA cm^−2^ and 1 mAh cm^−2^ due to the improvement of the lithiophilicity. After the infusion of molten lithium, an alloying reaction occurs, the principle of which is shown in Equation (1).

(1) $$2{\mathrm{Cu}}_{41}{\mathrm{Sn}}_{11}+55\mathrm{Li}\to 11{\mathrm{Li}}_{5}{\mathrm{Sn}}_{2}+82\mathrm{Cu}$$

The resulting LiSn alloy can promote uniform Li deposition and the rapid migration of Li-ions. The 3D porous skeleton can suppress dendrite growth and alleviate the volumetric expansion of the electrode. As a result, the 3D Cu–LiSn–Li||LFP full cell exhibited a superior cycling performance at 5 C, as presented in Figure 2c. Moreover, the 3D Cu–LiSn–Li||LFP full cell showed a better rate performance than the Bare Li||LFP full cell at various rates (Figure 2d).

In addition to chemical etching and vapor dealloying, electrochemical etching is also investigated for use in the preparation of 3D Cu CCs. For instance, Zhao et al. fabricated a 3D porous Cu CC as a Li host via the electrochemical etching of copper–zinc alloy [70]. The as-prepared uniform and compact 3D porous structure not only has high electrical conductivity and mechanical properties, but also helps to form a smooth and stable SEI. The excellent properties and suitable porous structure of the 3D Cu can effectively inhibit lithium dendrites and dead lithium from arising. Consequently, the Li@3D Cu||LiFePO_4_ full cells exhibited superior cycling and rate performance. Similarly, Li and coworkers reported a three-dimensional hierarchical porous copper (3DHP Cu) CC produced via an electrochemical dealloying method [71]. The preparation process of 3DHP Cu is shown in Figure 2e. After dealloying, a homogeneous and compact porous structure can be observed on the Cu surface, as presented in Figure 2f. The hierarchical distribution of micropores and nanopores (500–800 nm) can promote the migration of Li-ions, make the current distribution uniform, and alleviate the volume change. The symmetric cells based on 3DHP Cu could stably cycle for more than 250 h at 3 mA cm^−2^ and 1 mAh cm^−2^ with a low overpotential (Figure 2g). Moreover, the Li@3DHP Cu||LiFePO_4_ full cell exhibited superior rate capability, as shown in Figure 2h. The outstanding electrochemical performances can be attributed to enhanced electrode reaction kinetics and uniform Li plating, because the hierarchical distribution of micropores and nanopores provides rich lithium-ion rapid transport channels.

In summary, dealloying is a facile method for preparing 3D Cu-based CCs. Various dealloying techniques have their own characteristics. Chemical dealloying often uses acid and alkali solutions as etching solutions, which is not friendly to the environment. Hence, it is necessary to study green etchants as part of the development of chemical dealloying technology. In contrast, electrochemical dealloying usually uses salt solutions and has less impact on the environment. However, the effect of electrochemical dealloying is affected by many factors, including voltage, current, time, temperature, etc. Therefore, it is important to explore the interaction between different factors. The vapor dealloying process is simple and friendly, but it is limited by the melting and boiling points of the alloy components, and the energy consumption is high in some cases.

#### 2.1.3. Electrodeposition

Electrodeposition involves the formation of a coating through the transfer of positive and negative ions within an electrolyte solution, induced by an external electric field. This process involves oxidation-reduction reactions on the electrode, resulting in the gain and loss of electrons. Electrodeposition is also widely used in the preparation of 3D CCs.

**Figure 3:**
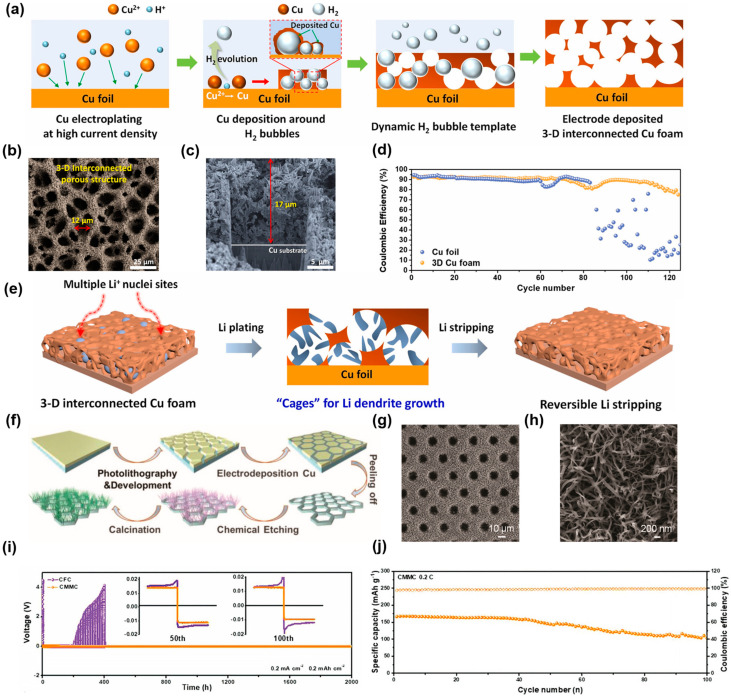
(**a**) Schematic illustration of the method of synthesizing 3D interconnected porous Cu foam. (**b**) SEM image of 3D Cu foam. (**c**) FIB–SEM image of 3D interconnected porous Cu foam. (**d**) Cycling performance of 3D Cu foam. (**e**) Li nucleation and plating/stripping behavior on 3D Cu foam electrode. Reprinted with permission [73]. Copyright 2023, Elsevier. (**f**) Process of synthesizing CMMC. (**g**) SEM images of CMMC. (**h**) SEM images of CMMC. (**i**) Cycling performances of Li–CMMC and Li–CFC electrodes. (**j**) Cycling stability of CMMC–LiǀǀLFP battery at 0.2 C. Reprinted with permission [74]. Copyright 2023, Wiley-VCH.

One of the more commonly used methods for preparing 3D copper structures is to electroplate copper on the planar Cu substrate. However, the deposited copper is easily fractured from the substrate due to its brittleness. In view of this, Volder and coworkers fabricated 3D Cu–CNT composites with mechanically resilient structures via co-plating carbon nanotubes (CNTs) with Cu on the copper substrate [72]. The 3D Cu–CNT CC with an open porous structure and suitable specific surface area can accommodate the plating of Li and avoid the excessive consumption of electrolytes caused by the introduction of CNTs. Moreover, the 3D Cu–CNT composites can also be calendered without damaging the structure, which has great potential in relation to improving the specific energy of LMBs. In addition, to inhibit the growth of lithium dendrites and unstable surface reactions, Kim et al. proposed an “Li dendrite cage” strategy and prepared a 3D interconnected porous Cu foam CC for LMA via a simple electrodeposition method [73]. Figure 3a shows the procedure of fabricating the 3D interconnected porous Cu foam. The surface morphology of the 3D Cu foam is presented in the SEM image (Figure 3b). With the assistance of a dynamic hydrogen bubbles template, three-dimensional porous structures with an average pore diameter of 12 μm were successfully fabricated on copper foil. The thickness of the 3D Cu foam is ~17 μm, as presented in the cross-sectional SEM (Figure 3c). Figure 3d shows the CE performance of 3D Cu foam and Cu foil at 0.5 mA cm^−2^, which exhibits that the CE of 3D Cu foam is more stable than the Cu foil. This can be ascribed to the advantage of the “cage effect”, that is, the dendrite’s growth is restricted within the abundant inner pores, inhibiting its growth to the outside, as shown in Figure 3e. Meanwhile, volume changes during the cycles are also mitigated due to the sufficient space within 3D structures. In addition, Zhang et al. prepared an ultrathin 3D array-structured Cu current collector via electrodeposition [74]. Firstly, the patterned design is established, and then the copper is electrodeposited to obtain the 3D current collector, as presented in Figure 3f. The surface morphology of the Cu CC can be observed in Figure 3g, h. This current collector (referred to as CMMC) has a low areal density, making it ultra-thin and lightweight, which helps in the construction of high-energy-density LMBs. Figure 3i shows the cycling performance of symmetrical cells at 0.2 mA cm^−2^ and 0.2 mAh cm^−2^. The Li–CMMC electrode exhibited a stable cycling over 2000 h at a low polarization voltage of 12 mV. Due to the synergistic effect of lithiophilic Cu_x_O and its appropriate structural design, the full cells with CMMC exhibited good cycling performance. Furthermore, as shown in Figure 3j, the capacity retention rate of the CMMC–Li||LiFePO_4_ full cell can reach 71% after 100 cycles.

#### 2.1.4. Others

Apart from the several methods mentioned above, some other works have been reported. For example, Zhang et al. obtained an ultrathin hierarchical porous Cu CC through the anodic oxidation method [75]. The uniform 3D micro/nanopores could effectively homogenize the local electric field and induce uniform Li deposition, thereby suppressing the Li dendrites’ growth and forming a stable SEI layer. As a result, the full cell’s performance with this current collector is improved. As a material preparation technology that has developed rapidly in recent years, the application of 3D printing in the field of energy storage, especially in rechargeable batteries, has been widely investigated. For example, Lei and coworkers reported a 3D Cu mesh produced by 3D printing [76]. Unlike the uneven electric field on the surface of the traditional Cu CC, the 3D-printed structures can effectively modulate the electric field distribution and provide sufficient internal space for Li deposition. The subsequent electrochemical properties also indicate that the 3D Cu mesh could suppress Li dendrite growth, improve CE, and mitigate volume changes, which shows the great potential of using 3D printing in the preparation of a 3D current collector.

### 2.2. Chemical Modification

In addition to designing or adjusting the structure of the current collector, other materials can also be introduced to improve the surface properties of the Cu CCs. This class of methods can be classified as chemical modifications, which include functional spot modification [77,78], oxidation modification [79,80], protective layer modification [81] and so on.

#### 2.2.1. Functional Spot Modification

Previous studies have shown that lithium has a large nucleation overpotential on a copper substrate, which indicates that copper’s surface is lithiophobic [82]. Therefore, improving the surface properties of Cu CC and enhancing its lithium affinity can promote uniform Li deposition and improve the cyclic stability of LMBs.

**Figure 4:**
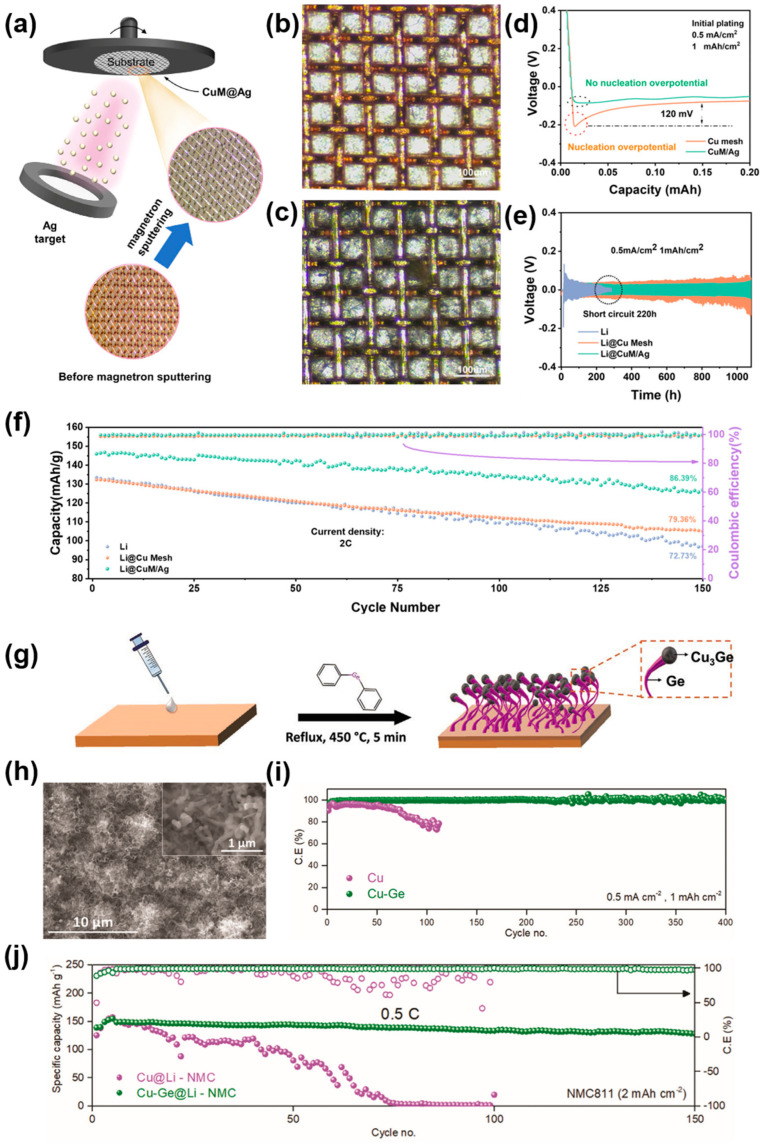
(**a**) Schematic diagram of method of synthesizing CuM/Ag. (**b**) Digital photos of CuM/Ag under optical microscopy. (**c**) Digital photographs of Li@CuM/Ag. (**d**) Voltage curves of Li plating on Cu mesh and CuM/Ag. (**e**) Cycling performance of Li, Li@Cu mesh, and Li@CuM/Ag electrodes. (**f**) Cyclic stability of Li@CuM/Ag||LCO cell at 2 C. Reprinted with permission [84]. Copyright 2023, Wiley-VCH. (**g**) Schematic diagram of Ge NWs synthesis on Cu foil. (**h**) SEM image of Cu-Ge. (**i**) Electrochemical performance of the Gu–Ge. (**j**) Cycling stability of Cu–Ge@Li–NMC811 battery at 0.5 C. Reprinted with permission [91]. Copyright 2023, Wiley-VCH.

Cui and coworkers found that some metals (such as Au, Ag, Zn and Mg) can form alloys with lithium to reduce nucleation overpotential and induce uniform Li deposition [82]. For instance, Han and coworkers introduced lithiophlic Ag nanoparticles (Ag NPs) onto graphene sheets as lithium hosts, achieving the uniform deposition of lithium [83]. Similarly, these metals can be employed to modify the copper current collectors, thereby augmenting the lithiophilicity of the Cu-based current collectors. For example, Chen et al. prepared silver-modified copper mesh as a current collector via the magnetron sputtering method [84]. The process of fabricating Cu mesh with Ag layer (CuM/Ag) is shown in Figure 4a. The lithiophilic Ag layer is uniformly distributed on the Cu framework (Figure 4b). Figure 4c shows the structure of the Cu/Ag/Li composite anode (Li@CuM/Ag) under an optimal microscope. As shown in Figure 4d, CuM/Ag exhibited negligible nucleation overpotential due to the excellent lithiophilicity of CuM/Ag. The silver layer could effectively reduce nucleation overpotential and induce uniform lithium deposition at the nucleation sites. The Li-Ag alloy produced by the reaction of the silver layer with lithium shows better reversibility during the process of Li deposition/stripping and interfacial reaction kinetics. Hence, Li@CuM/Ag symmetric cells can stably cycle over 1000 h at 0.5 mA cm^−2^ and 1 mAh cm^−2^, and the overpotential is only 25 mV, as shown in Figure 4e. Figure 4f shows the cyclic stability of full cells. At the rate of 2 C, the initial specific capacity (146 mAh g^−1^) and the capacity retention rate (86.39% after 150 cycles) of the Li@CuM/Ag||LiCoO_2_ full cells are higher than those of Li@Cu mesh||LiCoO_2_ and Li||LiCoO_2_ full cells. The excellent cycling performances can be attributed to the enhanced lithiophilicity and uniform Li deposition because of the introduction of a silver layer onto the Cu mesh. In addition to some commonly used lithiophilic metals, such as zinc, silver, etc., the application of some metals (such as bismuth, tungsten, gallium, germanium, vanadium, etc.) and their compound materials in the field of energy storage is gradually being explored [85,86,87,88,89,90]. For instance, Geaney and coworkers reported a novel Germanium (Ge) nanowires (NWs)-modified 3D Cu-based current collector [91]. The synthesis procedure is shown in Figure 4g. The thermal decomposition of diphenyl germane (DPG) stimulates the growth of Ge NWs from a copper germinide (Cu_3_Ge) seed. Such Ge NWs grows directly on the surface of copper without binders or conducting agents, helping to improve the energy density of LMBs. Figure 4h shows the morphology of the Cu–Ge surface. Ge NWs can be observed to grow densely on the surface of copper. Densely grown Ge NWs have high lithiophilicity and can provide abundant lithiophilic anchoring sites, which is conducive to regulating lithium-ion flux, lowering the local current density, and facilitating homogeneous Li plating. As a consequence, the Cu–Ge CC exhibited stable cycling (>400 cycles) and a high average CE (99.2%) at 0.5 mA cm^−2^ and 1 mAh cm^−2^, as shown in Figure 4i. Moreover, the Cu–Ge@Li–NMC full cell with a high-voltage NMC811cathode can cycle steadily for 150 cycles at 0.5 C with almost no capacity loss, as presented in Figure 4j. The superior electrochemical properties can be ascribed to the novel 3D structure and excellent lithiophilicity due to the introduction of Ge NWs.

#### 2.2.2. Oxidation Modification

In addition to introducing lithiophilic sites, copper oxides (CuO and Cu_2_O) have been demonstrated to be beneficial in promoting the uniform deposition of lithium. This is because copper oxides can improve the lithiophilicity of Cu-based CCs, promote the transport of Li-ions and enhance the stability of SEI by the in-situ formation of Li_2_O with Li. This method of oxidizing copper to obtain copper oxides, thereby improving the lithiophilicity of the CCs and facilitating homogeneous Li deposition, is called the oxidation modification. The most commonly used oxidation methods include electrochemical anodizing [92], chemical oxidation [79], thermal oxidation [93], etc.

**Figure 5:**
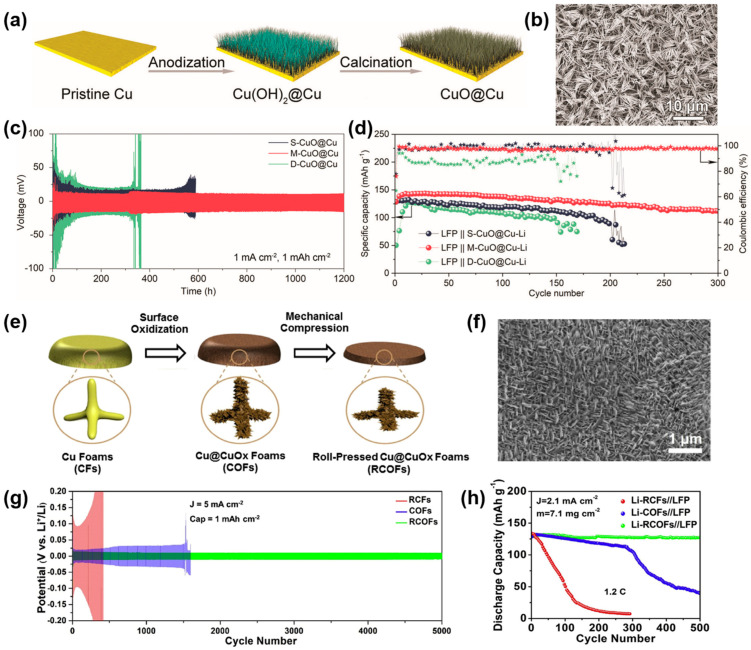
(**a**) Process of synthesizing CuO@Cu nanowire arrays. (**b**) SEM image of the surface view of M–CuO@Cu nanowire arrays. (**c**) Cycling stability of CuO@Cu–Li anodes in symmetric cells. (**d**) Cycling performance of LFP||CuO@Cu–Li full-cells at 1 C. Reprinted with permission [92]. Copyright 2023, Wiley-VCH. (**e**) The fabrication of roll-pressed Cu@CuO_x_ foams (RCOFs). (**f**) SEM image of RCOFs. (**g**) Cycling performances of Li–RCOFs electrode. (**h**) Cycling performances of Li–RCOFs//LFP cell. Reprinted with permission [94]. Copyright 2020, Elsevier.

The oxidation treatment of copper foil is a facile method used to construct three-dimensional current collectors based on planar copper. At the same time, copper oxides can also enhance the lithiophilicity of the Cu substrate. For instance, Liu et al. fabricated a 3D integrated gradient Cu-based CC via electrochemically anodizing [92]. The 3D structure is achieved by growing copper oxide (CuO) nanowire arrays on the copper foil, and the synthesis procedure is shown in Figure 5a. The morphology of CuO nanowire arrays is shown in Figure 5b. The density of the CuO nanowire arrays has a great influence on the Li deposition behavior, which depends on the anodizing time. According to the different anodizing times, the prepared current collectors can be divided into S–CuO@Cu (anodizing for 100 s), M–CuO@Cu (anodizing for 500 s), and D–CuO@Cu (anodizing for 1000 s). Sparse nanowire arrays (S–CuO@Cu) expose a considerable amount of the lithiophobic surface and cannot inhibit Li dendrite growth. Excessively dense nanowire arrays (D–CuO@Cu) hinder the downward deposition of lithium ions due to the blockage of channels during Li nucleation, causing the top deposition of lithium. Only the uniform nanowire arrays (M–CuO@Cu) can induce the bottom-up deposition of lithium and inhibit the generation of Li dendrites. Therefore, as shown in Figure 5c, M–CuO@Cu–Li exhibited excellent cyclic stability with a low voltage hysteresis for more than 1200 h at 1 mA cm^−2^ and 1 mAh cm^−2^. Moreover, the LFP||M–CuO@Cu–Li full cell displayed excellent cyclic performance, with a capacity retention rate of approximately 88% after 300 cycles at 1 C, and the full cell maintained high and stable Coulombic efficiency simultaneously (as shown by red stars), as presented in Figure 5d. The excellent cycling performances can be attributed to the reasonable nanowire arrays structures and enhanced lithiophilicity. It is also proven that a reasonable three-dimensional structure is of significance for uniform lithium deposition.

In addition to copper foil, the 3D Cu (such as copper foam, copper mesh) can also be modified by oxidation. For example, Qian and coworkers reported the production of pressure-tuned and surface-oxidized copper foams (RCOFs) via chemical oxidation and mechanical compression [94]. The preparation procedure is shown in Figure 5e. After mechanical rolling, RCOFs still maintained their 3D structure, and the morphology is shown in Figure 5f. Cu_x_O improves the lithiophilicity of copper foam, and the pore structure of copper foam is regulated by mechanical roller pressing. The synergistic effects of surface modification and structural regulation give RCOFs good electrochemical properties. The symmetric cells-based RCOFs can cycle for 2000 h with a low and stable polarization at 5 mA cm^−2^ and 1 mAh cm^−2^, as presented in Figure 5g. The Li–RCOFs//LFP full cell exhibited a high capacity for retention of 99% after 500 cycles at 1.2 C (Figure 5h), which shows the obvious superiority of this joint modification strategy. The outstanding electrochemical performances can be ascribed to the reasonable adjustment of the porosity and lithiophilicity of Cu foam by mechanical rolling and oxidation treatment.

#### 2.2.3. Protective Layer Modification

A major obstacle in the application of LMA is its high reactivity. The reaction of lithium with the electrolyte causes the consumption of the electrolyte and the loss of active Li, and the generated weak SEI is also not conducive to uniform Li deposition. To solve this problem, the strategy of constructing an artificial protective layer (or artificial SEI) on Cu-based CCs has been proposed and studied extensively.

**Figure 6:**
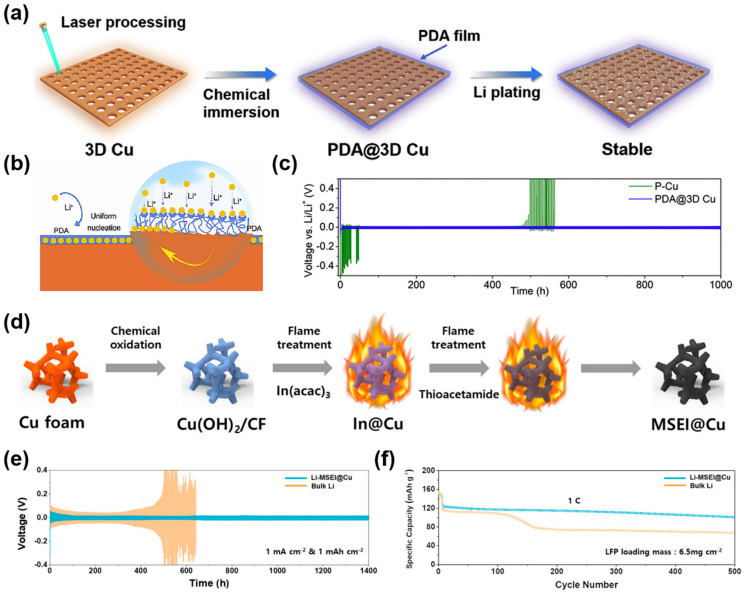
(**a**) Schematic diagram of the fabrication and lithiation process of PDA@3D Cu. (**b**) Schematic illustration of Li deposition through PDA layer. (**c**) Cycling performance of Li@PDA@3D Cu electrodes. Reprinted with permission [95]. Copyright 2020, Elsevier. (**d**) Process of synthesizing MSEI@Cu. (**e**) Cycling performances of Li–MSEI@Cu electrode. (**f**) Cycling stability of Li–MSEI@Cu||LFP cell at 1 C (1C = 170 mAh g^−1^). Reprinted with permission [97]. Copyright 2024, The Royal Society of Chemistry.

Organic materials have been widely studied for their use as modification layers on Cu-based CCs due to their good structural flexibility and interfacial compatibility with lithium metal. Furthermore, organic materials contain abundant polar functional groups. On the one hand, these functional groups can be adsorbed on the surface of the copper current collector through bonding or other interactions, avoiding the “tip effect”; on the other hand, polar functional groups can absorb Li-ions and improve the chemical affinity between Li-ions and the electrolyte, thus homogenizing lithium-ion flux and promoting uniform Li deposition. For example, Jiang et al. fabricated a 3D Cu-based CC modified with polydopamine (PDA) by laser processing and chemical treatment [95]. The preparation process is illustrated in Figure 6a. Laser processing provides a planar Cu-rich internal space and large specific surface area. Moreover, the PDA thin layer with abundant lithiophilic functional groups (such as –OH and –NH_2_) can effectively decrease the nucleation overpotential and facilitate uniform Li nucleation and deposition, as displayed in Figure 6b. Furthermore, the PDA layer can act as a strong artificial SEI layer to inhibit the growth of lithium dendrites and relieve volume expansion due to its excellent mechanical strength and toughness. Hence, the PDA@3D Cu electrode can cycle steadily for more than 1000 h with a low voltage hysteresis (~24 mV) at 0.5 mA cm^−2^ (Figure 6c).

Apart from organic protective layers, many inorganic materials have been investigated as protective layers of Cu-based CCs. For example, Liao and coworkers fabricated a Cu-based current collector modified with a Zn_3_N_2_ protective layer using filtered cathode vacuum arc (FCVA) technology [96]. When lithium ions are first deposited, the Zn_3_N_2_ protective layer reacts with lithium to generate LiZn alloy and lithium nitride (Li_3_N). The LiZn alloy enhances the lithiophilicity of the Cu CC and acts as the nucleating seed to induce uniform Li nucleation and deposition. Meanwhile, Li_3_N with high ion conductivity can be used as the artificial SEI layer to promote the transport of lithium ions and isolate the electrolyte and LMA. Therefore, the Zn_3_N_2_@Cu||LFP anode-free full cell exhibited a high-capacity retention rate of 63.1% after 100 cycles, which is significantly better than that of the Cu||LFP full cell (14.9% after 100 cycles). In addition, Piao and coworkers constructed a Cu-based 3D host modified with a multifunctional solid electrolyte interphase [97]. The procedure of synthesizing the modified 3D host (MSEI@Cu) is shown in Figure 6d. MSEI@Cu is prepared via a novel double-coating strategy. Specifically, Cu nanowires grown on a copper foam surface are covered by a double coating, which includes a compact surface carbon layer and an internal carbon matrix containing CuSO_4_ and In_2_S_3_. After the reaction with lithium, an artificial SEI layer rich in Li_2_S and Li_x_In is formed on the surface of MSEI@Cu. At the same time, the content of LiF is increased by facilitating the decomposition of TFSI^−^ anions. The rich inorganic components significantly improved the mechanical properties and stability of SEI, and enhanced the transport kinetics of Li-ions. The amorphous carbon layer on the surface can accommodate the volume change of the electrode and isolate the contact between the electrolyte and the inner layer, inhibiting the excessive decomposition of the electrolyte. Hence, the electrochemical properties of the Li–MSEI@Cu were significantly improved. The symmetric cell with a Li–MSEI@Cu electrode can stably cycle for 1400 h, with a low overpotential of 15 mV at 1 mA cm^−2^ and 1 mAh cm^−2^ (Figure 6e). Accordingly, the Li–MSEI@Cu||LFP full cell exhibited excellent cyclic stability for 500 cycles with 80% capacity retention at 1 C, as shown in Figure 6f. The excellent cycling performances can be attributed to enhanced lithium-ion transport kinetics and the inhibition of the excessive decomposition of the electrolyte due to the ingenious design of multifunctional SEI. It is worth noting that the novel modification method provides a new idea for constructing a multi-functional artificial SEI.

## 3. 3D Cu-Based CCs for SMBs and PMBs

Sodium metal anodes (SMAs) and potassium metal anodes (PMAs) are considered ideal alternatives to Li for use in next-generation high-performance batteries due to their high theoretical specific capacity and low cost. Similarly, as alkali metal anodes, they encounter the same problems and challenges as lithium metal anodes, such as dendrite growth, volume expansion, fragile SEI and so on [98,99,100,101]. Many research results have shown that 3D Cu-based current collectors can also be used in sodium metal batteries (SMBs) and potassium metal batteries (PMBs) to improve their performance.

**Figure 7:**
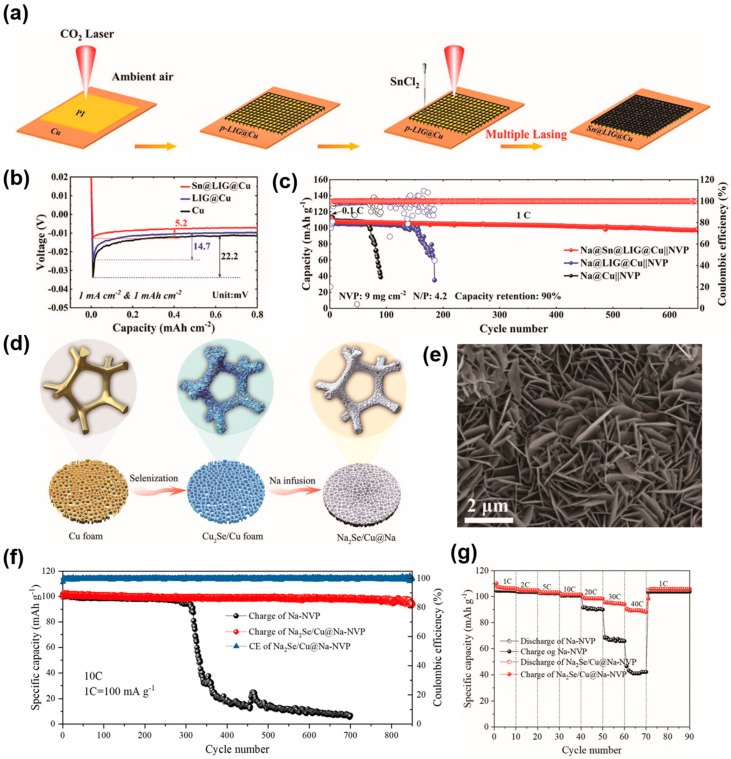
(**a**) Process of synthesizing Sn@LIG@Cu. (**b**) Voltage curves of Na deposition on different substrates. (**c**) The cycle performance of Na@Sn@LIG@Cu||NVP at 1 C. Reprinted with permission [105]. Copyright 2023, Wiley-VCH. (**d**) Synthesis procedure of Na_2_Se/Cu@Na composite anode. (**e**) SEM image of CF/Cu_2_Se. (**f**) The cycling performances of Na||NVP and Na_2_Se/Cu@Na||NVP full batteries at 10 C. (**g**) The rate capacity of Na_2_Se/Cu@Na||NVP full batteries. Reprinted with permission [106]. Copyright 2022, Wiley-VCH.

To date, 3D Cu-based current collectors have been explored for use in sodium metal anodes [102,103]. For example, Chen et al. prepared a Cu_6_Sn_5_ alloy layer on Cu foils (Cu_6_Sn_5_@Cu) and applied it to sodium metal anodes [104]. The sodiophilic Cu_6_Sn_5_ can significantly reduce the nucleation overpotential of Na. Besides this, Cu substrates can alleviate the volume and stress changes during alloying and maintain the structural stability of the current collectors. As a result, the Cu_6_Sn_5_@Cu CC showed high average CE (over 99.84%) during 2000 cycles at 5 mA cm^−2^ and 1 mAh cm^−2^. In addition, Huang’s group reported a facile approach to stabilizing the SMAs by constructing Sn nanoparticles-anchored graphene on planar Cu (Sn@LIG@Cu) [105]. Figure 7a illustrates the process of preparing the Sn@LIG@Cu CC. The Sn nanoparticles can improve the sodiophilicity of Cu current collectors and reduce the Na nucleation overpotential, as shown in Figure 7b. The low overpotential (~5.2 mV) is conducive to Na nucleation and dendrite-free sodium deposition. Based on this, the Sn@LIG@Cu showed a high average CE after a long cycle. Moreover, the flexible polyimide (PI) columns can act as the binder and buffer layer, which can effectively mitigate the volume change of SMAs during cycling. The unique patterned structure design provides continuous channels for rapid ion transportation, thus promoting the Na-ions’ transport kinetics. In view of this, the Na@Sn@LIG@Cu||NVP full cell exhibited superior cycling stability over 600 cycles with 90% retention capacity at 1 C (Figure 7c). The excellent electrochemical performances can be ascribed to enhanced Na^+^ transport kinetics and dendrite-free sodium deposition due to their unique patterned structure and the introduction of sodiophilic Sn nanoparticles. Moreover, this unique structure design and advanced preparation method provide a feasible approach to the application of 3D Cu-based CCs on SMAs. When applied to alkali metal batteries, the Cu-based current collector has the problem of poor affinity with alkali metals. Therefore, the surface modification of Cu-based CCs has been widely investigated. For instance, Yu and coworkers fabricated a porous Cu skeleton modified with cuprous selenide nanosheets (Cu_2_Se/Cu foam) via the selenization treatment of Cu foam [106]. The composite SMA (Na_2_Se/Cu@Na) is prepared by infusing molten Na into a Cu_2_Se/Cu foam, as shown in Figure 7d. The Cu_2_Se nanosheets vertically grown on the surface of copper foam play crucial roles in improving the Na metal anode’s performance (Figure 7e). On the one hand, the uniform distribution of Cu_2_Se nanosheets can improve the sodiophilicity of Cu CC and promote the infusion of molten sodium, thus forming a composite anode, whereas the bare copper foam cannot be wetted by molten sodium to form a composite SMA. On the other hand, the Na_2_Se nanosheet clusters formed after molten sodium infusion can promote the rapid transport of sodium ions and enhance the electrode reaction kinetics. Meanwhile, the 3D composite structure can homogenize the distribution of Na-ion flux, inhibit the volume change, and facilitate uniform Na deposition. As a result, the Na_2_Se/Cu@Na||NVP (Na_3_V_2_(PO_4_)_3_) full cell delivered an initial charge capacity of 102 mAh g^−1^ with 95.1% capacity retention after 800 cycles, even at 10 C, as presented in Figure 7f. Moreover, as shown in Figure 7g, compared with bare Na||NVP full cells, the Na_2_Se/Cu@Na||NVP full cells exhibit a higher discharge capacity, especially at high rates. Such excellent performances can be attributed to the fast sodium-ion transport and the stable three-dimensional structure. It is worth noting that the current collector is also applicable to potassium metal batteries, which indicates the excellent performance and great application potential of the 3D Cu-based CCs.

**Figure 8:**
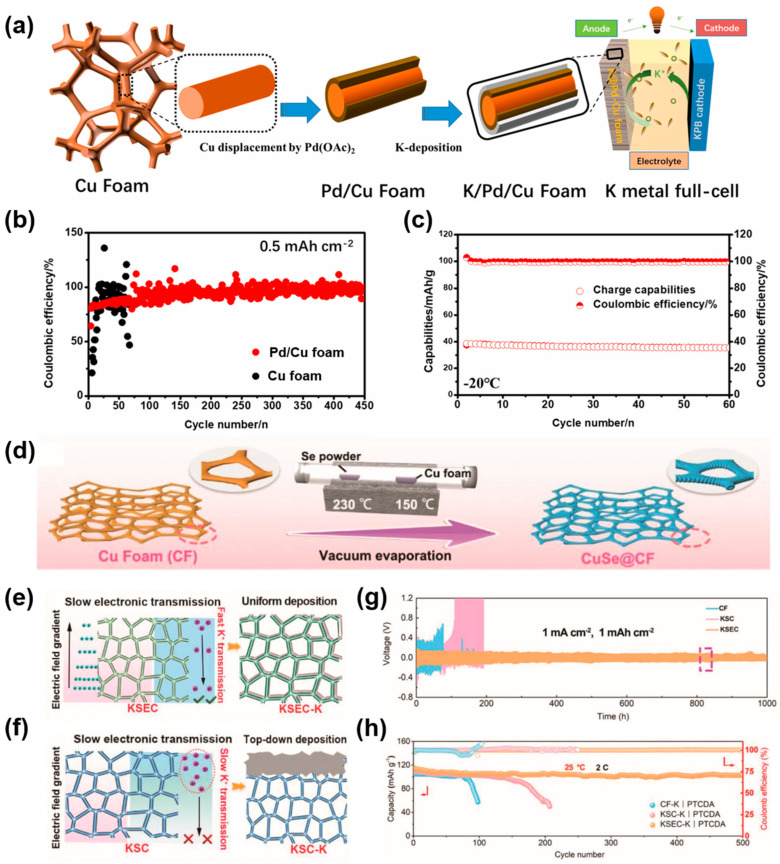
(**a**) Process of synthesizing Pd/Cu foam and K/Pd/Cu foam. (**b**) Electrochemical performance of the Pd/Cu foam. (**c**) Cycling stability of K/Pd/Cu||PB cell at −20 °C. Reprinted with permission [109]. Copyright 2022, Elsevier. (**d**) Schematic diagram of the synthesis procedure of CuSe@CF. (**e**) K deposition behavior on KSEC anode, “synchronized” deposition. (**f**) K deposition behavior on KSC anode, “top-down” depositional. (**g**) Galvanostatic voltage profiles of KSC and KSEC electrodes. (**h**) Cycling performance of KSEC–K|PTCDA cell at 2 C. Reprinted with permission [111]. Copyright 2023, Wiley-VCH.

Apart from SMAs, 3D Cu-based CCs have also been investigated for PMAs [107,108]. For instance, Wang et al. designed a Cu_3_Pt alloy-modified Cu mesh and applied it as the CC in PMBs [108]. The Cu_3_Pt-Cu mesh has a coarse surface with massive nanoparticles, which can provide a large specific area and homogenize the distribution of electrical field and ion flux. Moreover, Cu_3_Pt has excellent affinity for K, which can lower the nucleation overpotential and induce uniform K deposition. Accordingly, the full cell (Prussian blue (PB) as the cathode material, Cu_3_Pt-Cu mesh as the anode CC) exhibited an ultralong lifespan over 250 cycles. Similarly, Zhang’s group prepared a highly potassiophilic Pd/Cu CC, and investigated the application in a low-temperature K metal battery [109]. Figure 8a shows the fabrication process of the Pd/Cu current collector, K/Pd/Cu anode and the cell configuration. The coated Pd layer enhances the potassiophilicity of the CC, and the Cu foam with large specific surface area could lower the local current density, which enables the Pd/Cu current collector to exhibit excellent electrochemical performance. The Pd/Cu shows a more stable CE than bare Cu foam over 450 cycles at 0.5 mA cm^−2^ and 0.5 mAh cm^−2^ (Figure 8b). Moreover, the K/Pd/Cu||PB full cell can stably cycle over 60 cycles even at −20 °C due to its excellent dendrite inhibition ability, as displayed in Figure 8c. Due to the huge differences in the transport speeds of electrons and ions on the three-dimensional current collector, electrons are prone to accumulate at the top of the current collector and form current hot spots, which makes the growth of dendrites inevitable. Hence, surface modification has been shown to regulate the electron and ion transport of the current collector [110]. For example, Zhao and coworkers demonstrated an up-and-down “simultaneous” deposition model in low-temperature and dendrite-free PMBs [111]. They introduced copper selenide (CuSe) to the surface of the Cu foam through vacuum evaporation (Figure 8d). Afterward, a K_2_Se/Cu (KSEC) conductive layer was obtained by reacting with potassiuam. The KSEC layer has low electron conductivity and can form an electric field gradient, thereby avoiding the accumulation of electrons and the formation of current hot spots. Meanwhile, the KSEC layer has excellent potassium ion transport kinetics, which could promote the rapid transfer of potassium ions from the top to the bottom for nucleation and deposition at the bottom. This strategy of simultaneously regulating the electronic and ionic conductivity of the 3D anode promotes uniform potassium deposition. Figure 8e shows the functional mechanism of KSEC. However, K_2_S/Cu (KSC) prepared by the same method follows a top-down deposition model owing to the slow diffusion of potassium ions in the K_2_S layer and the short diffusion distance, as presented in Figure 8f. Due to the reasonable structural and functional design, the KSEC electrode exhibited a long cycle lifespan over 1000 h with a low overpotential of 80 mV at 1 mA cm^−2^ and 1 mAh cm^−2^, as shown in Figure 8g. Moreover, the KSEC–K|PTCDA full cell exhibited an excellent cycling stability over 500 cycles with a high-capacity retention at 2 C (Figure 8h). The superior cycling performances can be ascribed to the reasonable adjustment of the electronic and ionic conductivity via the formation of the KSEC layer. And more details about the electrochemical performances of 3D Cu-based CCs in AMBs can be found in Table 1.

## 4. Conclusions and Outlook

**Figure 9:**
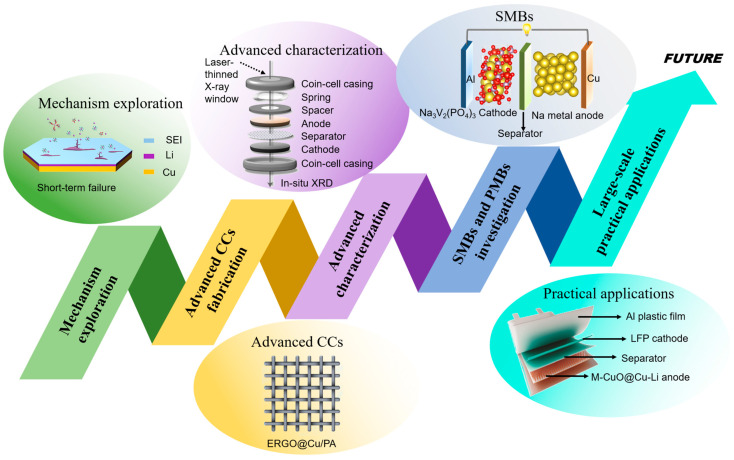
Outlook and prospective research directions relating to advanced 3D Cu-based current collectors for AMBs. Top left: reprinted with permission from [122] Copyright 2021, American Chemical Society. Top middle: reprinted with permission from [19] Copyright 2021, Nature Portfolio. The Royal Society of Chemistry. Top right: reprinted with permission from [123] Copyright 2024, The Royal Society of Chemistry. Bottom left: reprinted with permission from [124] Copyright 2023, American Chemical Society. Bottom right: reprinted with permission from [92] Copyright 2023, Wiley-VCH.

In conclusion, this review summarizes the commonly used methods for the modification of Cu-based CCs and recent progress on the applications of 3D Cu-based CCs for AMBs. The ideal three-dimensional Cu-based current collector would provide multiple advantages, which include (1) alleviating electrode volume changes; (2) reducing local current density and delaying dendrite growth; (3) reducing nucleation energy barriers; (4) changing SEI composition and promoting uniform deposition; (5) inducing the preferential deposition of alkali metal ions at the bottom. However, most of the existing current collector modification strategies struggle to take these points into account. As a result, appropriate modification strategies should be combined with the characteristics of structural modification and chemical modification to improve the performance of AMBs. In recent years, significant progress has been made in research into the application of 3D Cu-based CCs in AMBs. Nonetheless, there are still many challenges and problems that need to be solved. To better improve the performance of AMAs, further investigations should focus on the following points (Figure 9).

1. The mechanisms of the nucleation and growth of alkali metal ions and dendrite formation should be further investigated. For alkali metal batteries, the nucleation and growth behaviors of alkali metal ions determine the performance of the battery to a certain extent. Meanwhile, the dendrite problem, as a critical factor affecting the stability and safety of alkali metal batteries, has received much attention and research. Many current collector modifications are also aimed at inhibiting dendrite growth. At present, many works have been reported on promoting the uniform deposition of alkali metal ions and inhibiting dendritic growth. However, the explanation of the mechanism remains relatively singular, mainly regarding the reduction of local current density and the increases in nucleation sites. The existing modifications generally increase the specific surface area by constructing three-dimensional porous structures, thus reducing the local current density. These modification strategies can only delay the growth of dendrites to a certain extent, and cannot truly address the problem of dendrites. Therefore, the further exploration of more core mechanisms is essential to more effectively guide the development of Cu-based CCs and alkali metal batteries.

2. More advanced 3D Cu-based current collectors should be explored and investigated. Pure metal (pure Cu and Cu alloys)-based current collectors play a positive role in optimizing the performance of AMAs. However, these current collectors still face the problems of dendrite growth and volume change during long-term cycling, meaning they struggle to meet the actual application needs of alkali metal batteries. It is worth noting that some materials also play a huge role in the construction and modification of 3D Cu-based current collectors, including carbon materials, polymer materials and so on. Carbon materials (including graphite, graphene, etc.) have high electrical conductivity, excellent mechanical strength, light weight and so on [125]. It has been confirmed that constructing composite materials with carbon materials is a common method to improve the performance of electrode materials [126]. Polymer materials with polar functional groups have good flexibility and play a significant role in inducing lithium deposition and mitigating volume changes. In order to maximize the specific energy of AMBs, lightness of weight is the future development direction of current collectors. In view of the abundant reserves, low price and good electrical properties of copper materials, the study of lightweight three-dimensional copper composite current collectors (with carbon materials, polymer materials, etc.) is still of positive significance for the practical application of AMAs.

3. More advanced characterization techniques should be further explored and applied in alkali metal batteries. Compared with traditional characterization techniques (such as X-ray diffraction (XRD), scanning electron microscopy (SEM), transmission electron microscopy (TEM) and so on), advanced characterization techniques can more accurately measure the changes in various components and parameters in the battery system, thereby helping to clarify the relationship between various parameters. Undoubtedly, this helps to clarify the mechanisms by which battery performance improvement or battery failure occur. For example, in situ characterization techniques, including in situ XRD, in situ X-ray photoelectron spectroscopy (XPS), in situ Raman spectroscopy and in situ atomic force microscopy (AFM), etc., have developed rapidly in recent years, and have been widely used in the characterization of solid-state electrolytes. These characterizations can also be further explored and introduced into the study of current collectors.

4. The study of SMBs and PMBs should be further developed. As a topic of great interest, there are a lot of studies on lithium metal anodes, whereas there is little research on sodium metal anodes and potassium metal anodes. Although sodium, potassium and lithium encounter dendrite growth, volume change and other problems, there are some differences in their practical applications, including as cathode materials, electrolytes and so on. Besides this, in recent years, the lithium resource reserves have decreased and their price has risen, resulting in the high cost of lithium batteries. As a result, more attention and investigations should be directed towards SMBs and PMBs, which may replace lithium metal batteries in some applications in order to meet people’s demand for energy density.

5. The practical and large-scale application of 3D Cu-based current collectors in AMBs should be further investigated. At present, some studies on the application of 3D Cu-based CCs in alkali metal batteries have made great progress. However, their preparation methods or modified materials are not suitable for use in practical application. For example, some studies use magnetron sputtering, laser etching or other processes, and the equipment is expensive and not suitable for large-scale preparation. Besides this, some studies have used gold, silver or their compounds to improve the surface properties of Cu-based current collectors and regulate the nucleation and growth of alkali metals. Obviously, these expensive materials are not suitable for use in practical preparation and applications. In addition, most of the 3D Cu-based current collectors are assembled in coin cells to test their performance, which is different from the actual application environment. Hence, in this context, developing proper preparation methods and corresponding equipment is critical for preparing large-area 3D Cu-based CCs for use in practical AMBs.

Overall, this review summarizes some recent advances in the development of 3D Cu-based CCs for use in high performance AMBs. We hope this review will further promote the practical application of 3D Cu-based current collectors in AMBs and other fields.

## Figures and Tables

**Table 1:** Cycling performance of symmetrical cells under different 3D Cu-based current collector modification strategies.

Substrate	Current Collectors	Method	Cycle Performance V ^a^ (mV), T ^b^ (h) C_1_^c^ (mA cm^−2^), C_2_^c^ (mAh cm^−2^)	Ref.
**Lithium metal anodes**				
Modification strategy: Structrual modification				
/	3D Cu skeleton	Template method	40, 500 (1, 1)	[43]
Cu foil	Cu@Cu_x_O	Template method	20, 600 (1, 1)	[58]
Cu foam	HPC/CF	Template method	/, 620 (0.5, 1)	[60]
Cu-Zn alloy foil	3D Cu	Dealloying	/, 800 (0.52, 0.26)	[46]
Cu-Zn alloy foil	3D Cu	Dealloying	20, 400 (1, 1)	[70]
Cu-Zn alloy foil	2h-3D CuZn	Dealloying	25, 450 (1, 1)	[63]
Cu-Zn alloy foil	Porous Cu	Dealloying	20, 440 (1, 1)	[67]
Cu-Zn alloy mesh	HP-Cu@Sn	Dealloying Electroless plating	/, 800 (1, 1)	[112]
Cu foil	3DHP Cu	Electrodeposition Dealloying	33, 850 (1, 1)	[71]
Cu foil	3D P-CuZn	Electrodeposition Dealloying	/, 560 (1, 1)	[64]
Cu foil	3DOM Cu-450	Electrodeposition	25, 700 (0.2, 0.5)	[57]
Cu foil	Cu@Sn nanocones	Electrodeposition	10, 600 (1, 1)	[113]
Cu foil	3D Cu-CNT	Electrodeposition	/, 550 (0.5, 1)	[72]
/	3DP-Cu	3D printing	/, 250 (1, 1)	[114]
/	3D Cu mesh	3D printing	20, 500 (1, 1)	[76]
Modification strategy: Chemical modification				
Cu foam	Ag@CF	Chemical reaction	30, 1600 (1, 1)	[77]
Cu foil	Cu-Ge	Chemical reaction	/, 1000 (0.5, 1)	[91]
Cu mesh	CuM/Ag	Magnetron sputtering	25, 1000 (0.5, 1)	[84]
Cu foam	ISG-CuO-2mM	Chemical oxidation	/, 1150 (1, 1)	[79]
Cu foam	RCOFs	Chemical oxidation Mechanical rolling	/, 5000 (5, 1)	[94]
Cu foam Cu foam	Cu-Cu_x_O ZnO NFs/CuF	Chemical oxidation solvothermal	15, 1800 (1, 1) 10, 1600 (1, 1)	[115] [116]
Cu foil	CuO@Cu	Electrochemical anodizing	10, 1200 (1, 1)	[92]
Cu foam	GN@Cu foam	Chemical immersion	10, 2000 (0.5, 1)	[117]
Cu foil	PDA@3D Cu	Chemical immersion	24, 1000 (0.5, 0.5)	[95]
Cu foil	γ-APS-Cu	Drop casting	12, 1400 (0.5, 1)	[81]
Cu foil	GO-Zn/Cu	Electrodeposition Spin-coating	20, 600 (1, 1)	[118]
Sodium metal anodes				
Cu foam	CuNW-Cu	Electrochemical anodizing	25, 1400 (1, 2)	[102]
Cu-Zn alloy	3D porous Cu	Dealloying	/, 1000 (1, 1)	[103]
Cu foil	Cu/Zn/SnO_2_	Magnetron sputtering	25, 820 (1, 1)	[119]
Cu mesh	Pt-Cu/Cu mesh	Chemical reaction	/, 400 (1, 1)	[120]
Cu foam	SF-Cu-3.6	Chemical oxidation	19, 400 (1, 1)	[121]
Cu foam	Cu_2_Se/Cu foam	Solution selenization	70, 500 (1, 1)	[106]
Cu foil	Sn@LIG@Cu	Laser process	19.7, 1000 (10, 10)	[105]
Potassium metal anodes				
Cu foam	rGO@3D-Cu	Chemical immersion	/, 200 (0.5, 0.5)	[107]
Cu mesh	Cu_3_Pt-Cu mesh	Chemical reaction	1000, 300 (0.5, 1)	[108]
Cu foam	CuSe@CF	Vacuum evaporation	80, 1000 (1, 1)	[111]

^a^ Voltage hysteresis (mV); ^b^ time (h); ^c^ C_1_: current density (mA cm^−2^); C_2_: specific area capacity (mAh cm^−2^).

## Data Availability

The data are contained within this article.
